# Current Prognostic Biomarkers for Abdominal Aortic Aneurysm: A Comprehensive Scoping Review of the Literature

**DOI:** 10.3390/biom14060661

**Published:** 2024-06-05

**Authors:** Hamzah Khan, Mohamed Abu-Raisi, Manon Feasson, Farah Shaikh, Gustavo Saposnik, Muhammad Mamdani, Mohammad Qadura

**Affiliations:** 1 Division of Vascular Surgery, St. Michael’s Hospital, Toronto, ON M5B 1W8, Canada; 2 Li Ka Shing Knowledge Institute, St Michael’s Hospital, Unity Health Toronto, Toronto, ON M5B 1W8, Canada; 3 Division of Neurology, Department of Medicine, University of Toronto, Toronto, ON M5S 1A1, Canada; 4 Department of Surgery, University of Toronto, Toronto, ON M5T 1P5, Canada

**Keywords:** abdominal aortic aneurysm, vascular, biomarkers, protein, prognosis

## Abstract

Abdominal aortic aneurysm (AAA) is a progressive dilatation of the aorta that can lead to aortic rupture. The pathophysiology of the disease is not well characterized but is known to be caused by the general breakdown of the extracellular matrix within the aortic wall. In this comprehensive literature review, all current research on proteins that have been investigated for their potential prognostic capabilities in patients with AAA was included. A total of 45 proteins were found to be potential prognostic biomarkers for AAA, predicting incidence of AAA, AAA rupture, AAA growth, endoleak, and post-surgical mortality. The 45 proteins fell into the following seven general categories based on their primary function: (1) cardiovascular health, (2) hemostasis, (3) transport proteins, (4) inflammation and immunity, (5) kidney function, (6) cellular structure, (7) and hormones and growth factors. This is the most up-to-date literature review on current prognostic markers for AAA and their functions. This review outlines the wide pathophysiological processes that are implicated in AAA disease progression.

## 1. Introduction

The aorta is the largest artery within the human body, bearing the most pressure compared to any other artery [1]. The aorta is split based on its anatomical region into the thoracic aorta (consisting of the ascending aorta, the aortic arch, and the descending aorta) and the abdominal aorta (consisting of the suprarenal, and infrarenal aorta). The aorta ends within the lower abdomen, at the bifurcation where the common iliac arteries branch into each lower limb [1].

Aortic aneurysms are a disease characterized by the breakdown of the cellular elasticity of the aortic wall, leading to dilation and ballooning of the artery. Abdominal aortic aneurysms (AAAs), the most common type of aneurysm, are located within the abdomen, often below the renal arteries, and are considered aneurysmal when the artery dilates to 3 cm or larger, often exceeding more than 50% of the normal aortic diameter [2]. AAA can be a silent disease, with most patients having no symptoms. Diagnosis of this disease is vital, and if left untreated, AAAs can rupture, leading to severe life threatening internal bleeding, with a mortality of almost 90% [3].

AAA is often referred to as a non-specific disease, with a wide range of potential associated factors [4]. Generally, the causes of abdominal aortic aneurysm are due to a progressive destabilization of the extracellular matrix within the aortic wall in combination with inflammatory processes due to the migration of macrophages to the area. There is, however, not always a clear pathophysiological process, and it may not necessarily be associated with a single factor [5]. However, there are several risk factors known to be associated with AAA, including smoking, Caucasian ethnicity, a positive family history, hypertension, atherosclerotic disease, as well as increased age [6].

Patients with smaller AAAs (<5.5 cm) must be counselled for lifestyle changes, such as smoking cessation, and should begin medical management to control hypertension and hyperlipidemia to prevent the progression of the disease [2]. 

Physicians must make the decision of whether to conduct an open or endovascular aneurysm repair (EVAR) based on factors including the anatomical region and shape of the aneurysm, as well as the patient’s medical history, their risk of adverse cardiovascular events, risk of an endoleak, and patient’s ability to undergo a more intensive procedure. This decision can often be difficult. An endoleak occurs when blood leaks into the aneurysm sac outside the stent graft, which can happen if the graft does not completely seal the aneurysm or if there are small blood vessels still feeding the aneurysm sac. This complication is less common in open AAA repair because the aneurysm is directly removed and replaced with a synthetic graft, providing a more secure and permanent solution [7,8]. The decision-making process can be complex and requires a multidisciplinary approach. Though guidelines are available, such as that available through the Society for Vascular Surgery, as well as risk-scoring systems to aid in the decision-making process, there is no one-size-fits-all algorithm or calculation available. Each case is evaluated on its own merits, considering the most current clinical evidence, the surgeon’s expertise, and patients’ preference. 

Abdominal aortic aneurysm (AAA) is a complex disease that involves various biological pathways and processes, and understanding the progression of the disease can be difficult. Aneurysm size often may not be the only predictor of rupture or growth. Patients with aneurysms <5.5 cm may still be at risk of rupture and those with significantly larger aneurysms may never suffer any complications. Deciding whether to choose between open and endovascular repair can also be difficult. Therefore, finding predictive biomarkers for the prognosis of AAA at the time of diagnosis may be beneficial to risk stratify patients and monitor more closely those who are at a greater risk of complications and provide them with less invasive surgical strategies if required. 

In this in-depth literature review, we will focus on the current available prognostic biomarkers for AAA and categorize them based on physiological function. By identifying prognostic biomarkers for AAA, we hope to further understand the mechanisms behind the initiation of this disease, as well as set the groundwork for future research which may allow for further validation of these markers to be used in a clinical setting. With the current interest in the integration of machine learning models with biomarker analysis, we hope that this review can further provide information about potential markers that can be integrated into models for better risk stratification in patients with AAA.

## 2. Methods and Literature Review

**Figure 1:**
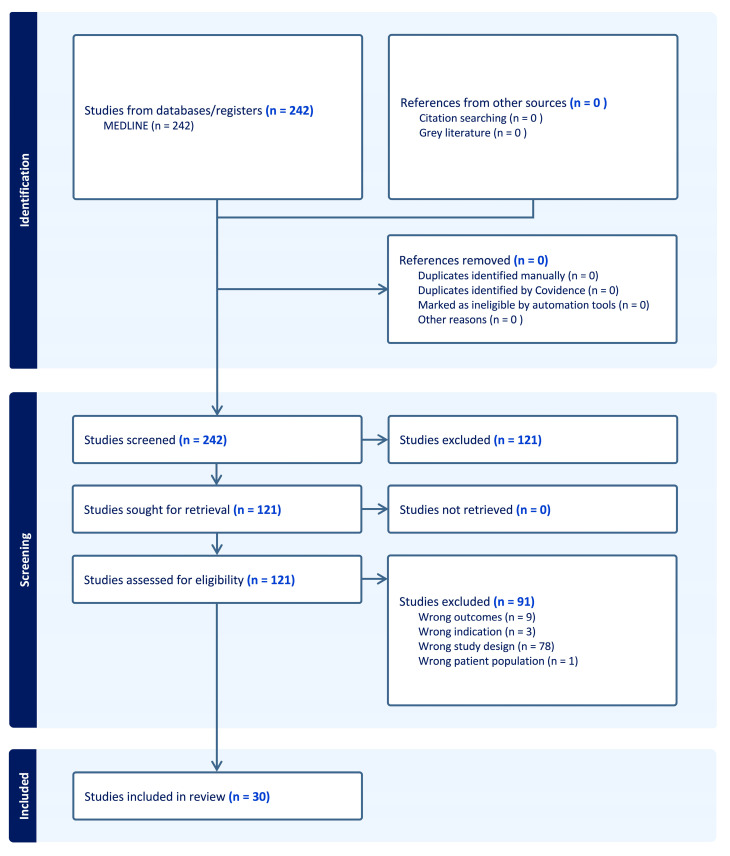
Preferred Reporting Items for Systematic Reviews and Meta-Analyses (PRISMA) diagram for MEDLINE search of biomarkers for the prognostication of abdominal aortic aneurysm. Diagram exported from Covidence (covidence.org).

The study is conducted following the PRISMA Statement, and the Review was not registered. A MEDLINE search was conducted for current research (January 2010–August 2023) investigating plasma and serum biomarkers for predicting AAA development, the need for AAA surgical repair, AAA-related deaths, and AAA-induced complications. “Abdominal Aortic Aneurysm” was cross-searched with “Protein” and “Biomarkers” in the MEDLINE database. Studies were filtered for English and human studies only. Abstract and title screening, followed by full paper review, was conducted by three individual reviewers on 242 studies after 35 non-human and 4 non-English papers were excluded (Figure 1). Papers that focused solely on diagnostic biomarkers, tissue biomarkers, non-protein markers, non-plasma or serum markers, thoracic aortic aneurysm markers, or post-surgical intervention markers only, were excluded from analysis (Figure 1). A total of 30 studies were included in this review (Table 1) with 45 proteins investigated for their potential as prognostic biomarkers for AAA. Proteins were then categorized based on their primary physiological functions. 

## 3. Prognostic Protein Categorization and Functions

**Figure 2:**
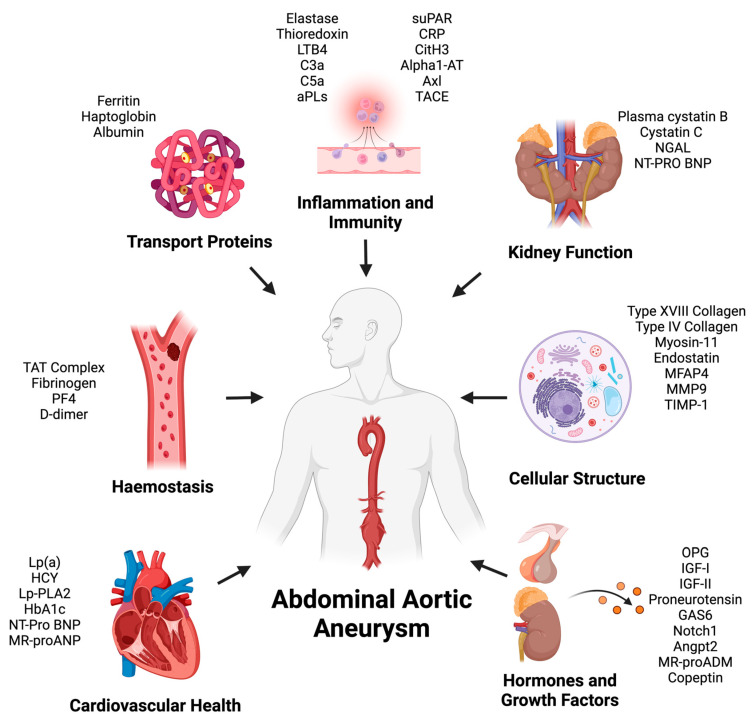
Current prognostic plasma/serum protein biomarkers for abdominal aortic aneurysm (AAA). Lipoprotein(a) (Lp(a)), homocysteine (HCY), lipoprotein-associated phospholipase A2 (Lp-PLA2), glycated hemoglobin (HbA1c), B-type natriuretic peptide (BNP), thrombin–antithrombin (TAT) complex, platelet factor 4 (PF4), microfibrillar-associated protein 4 (MFAP4), matrix metalloproteinase 9 (MMP9), TIMP metallopeptidase inhibitor 1 (TIMP-1), osteoprotegerin (OPG), insulin-like growth factor I (IGF-I), insulin-like growth factor II (IGF-II), growth arrest specific 6 (Gas6), Angiopoietin-2 (Angpt2), Leukotriene B4 (LTB4), Complement factor 3a (C3a), Complement factor 5a (C5a), antiphospholipid antibodies (aPLs), soluble urokinase plasminogen activator receptor (suPAR), C-reactive protein (CRP), citrullinated histone H3 (CitH3), Serpin Family A member 1 (alpha1-AT), tumor necrosis factor-α-converting enzyme (TACE), neutrophil gelatinase-associated lipocalin (NGAL), mid-regional proadrenomedullin (MR-proADM), and mid-regional pro-atrial natriuretic peptide (MR-proANP). This figure was created using BioRender (Biorender.com).

The proteins were classified into seven general categories based on their main function: (1) cardiovascular health, (2) hemostasis, (3) transport proteins, (4) inflammation and immunity, (5) kidney function, (6) cellular structure, (7) and hormones and growth factors (Figure 2).

### 3.1. Cardiovascular Health

Abdominal aortic aneurysm is a cardiovascular disease influenced by a myriad factors related to several general cardiovascular risk factors including increased age, smoking, hypertension, dyslipidemia, and family history of cardiovascular disease. Previous research has demonstrated that patients with concomitant coronary artery disease (CAD), or peripheral arterial disease (PAD), both atherosclerotic diseases, have an increased the risk of developing AAA (odds ratio for CAD of 2.29 (95% CI 1.75–3.01) and for PAD of 2.50 (95% CI 2.12–2.95)) [39,40,41]. Though AAA is not an atherosclerotic disease, atherosclerosis has been thought to contribute to the progression of the disease by releasing pro-inflammatory cytokines, shear stress, and arterial remodeling, which are hallmarks of atherosclerosis [42]. This is why regulation of atherosclerotic risk factors is important in patients with AAA; hence, biomarkers related to cardiovascular health have been a strong predictor of adverse aortic events. Below are four cardiovascular biomarkers that have been investigated as prognostic markers for AAA. 

#### 3.1.1. Homocysteine (HCY)

Homocysteine (HCY) is a methionine-derived, sulfur-containing, non-proteinogenic amino acid that is formed during the biosynthesis of other amino acids [43,44]. HCY levels can be elevated in patients with folate, Vitamin B_6_, and Vitamin B_12_ deficiencies, but can also be elevated due to genetic and other environmental factors [45]. Previous studies have demonstrated elevated levels of HCY in cardiovascular disease, indicating complications such as myocardial infarctions and cerebrovascular attacks [45]. One recent study sought to investigate the prognostic capability of HCY in predicting endoleak after EVAR [14]. Though patient with endoleaks post-EVAR had elevated levels of HCY compared to those without (16.0 (95% CI 11.3–19.2) mol/L versus 12.0 (95% CI 10.0–14.0) mol/L, *p* = 0.02, respectively), there was no significant association between HCY levels and endoleak post-EVAR.

#### 3.1.2. Lipoprotein-Associated Phospholipase A2 (Lp-PLA2)

Lipoprotein-associated phospholipase A2 (Lp-PLA2), previously known as platelet-activating factor acetylhydrolase, is a circulating enzyme linked to both low-density lipoprotein (LDL) and high-density lipoprotein (HDL), and is produced by circulating macrophages [46]. Its primary function is to hydrolyze oxidized phospholipids, forming lysophosphatidylcholine and oxidized free fatty acids [47]. It has been strongly linked to increased risk of atherosclerotic and cardiovascular disease, with increased levels of Lp-PLA2 being associated with an increased risk of MI, stroke, and cardiac death [48]. In this review, one research team investigated Lp-PLA2 levels in predicting AAA in a longitudinal study of 5551 patients. They discovered that Lp-PLA2 was independently associated with AAA development with a hazard ratio of 1.23 (95% CI 1.00–1.51, *p* = 0.049) after adjusting for confounding factors. This implied that elevated Lp-PLA2 suggested potential underlying susceptibility to AAA [26]. This may be due to the fact that Lp-PLA2 levels are strongly correlated with LDL and total cholesterol levels [49]. 

#### 3.1.3. Glycated Hemoglobin (HbA1c)

Hemoglobin is a vital carrier protein found within erythrocytes and carries oxygen throughout the body. Glycated or glycosylated hemoglobin (HbA1c) is a molecule that is formed due to a chemical reaction between plasma glucose and hemoglobin and is proportional to the levels of mean glucose within the blood between 8 and 10 weeks before the test [50]. It is used as a marker of long-term glucose control, with cut-off values >6.5% used as a marker of diabetes mellitus (DM) [51]. DM is a risk factor for several cardiovascular diseases, including atherosclerosis. Interestingly, diabetes mellitus is not a risk factor for AAA, and some studies have demonstrated a protective effect on aneurysmal growth [52,53,54,55,56]. One study investigated HbA1c levels in the growth of AAA and noted a similar trend, with there being a significant negative correlation between HbA1c levels and aneurysmal growth (Spearman ρ = −0.177; *p* = 0.002). After 3 years of follow-up, the highest tertile of HbA1c had a 1.5 mm (CI, 0.59–2.42; *p* = 0.001) smaller AAA compared to the lowest HbA1c tertile [20]. It is hypothesized that the glycosylation of collagen that is associated with DM may reduce the activity of matrix metalloproteinases, reducing the breakdown of collagen, thereby reducing the risk of AAA [57].

#### 3.1.4. B-Type Natriuretic Peptide (BNP) and N-Terminal proBNP (NT-proBNP)

B-type natriuretic peptide (BNP) is a peptide hormone released from cardiomyocytes, and is formed from the cleavage of the N-terminus of the proBNP peptide into BNP and N-terminal proBNP (NT-proBNP) [58]. BNP and NT-proBNP have become strong markers of cardiac function. BNP is released from cardiomyocytes in response to cardiac stretch, and leads to peripheral vasodilatation and an increase in sodium excretion and urine production. It also inhibits both the renin–angiotensin–aldosterone system to regulate blood pressure and electrolyte balance, and the sympathetic nervous system [59,60]. Both BNP and NT-proBNP are used clinically as a marker of heart failure [61]. There is currently one study that has investigated BNP as a prognostic marker for AAA, noting that patients with post-operative cardiac events (*p* < 0.001), as well as cardiac death (*p* < 0.043), had significantly elevated levels of BNP. BNP predicted post-operative cardiac events and cardiac death with high accuracy with a ROCAUC = 0.93 and 0.96, respectively [12]. Another study wanted to investigate the prognostic capabilities of NT-proBNP. Similarly, they noted that NT-proBNP was an independent predictor of MI with an AUC of 0.81, demonstrating good predictive capabilities [30]. Though these markers are not specific for aortic events, NT-proBNP and BNPa may be markers that can be associated with other cardiovascular outcomes in the AAA population.

#### 3.1.5. Mid-Regional Pro-atrial Natriuretic Peptide

Mid-regional pro-atrial natriuretic peptide (MR-proANP) is produced as a byproduct of atrial natriuretic peptide (ANP). ANP is a hormone released from cardiomyocytes in the right atrium in response to atrial stretch and leads to several changes such as natriuresis, diuresis, and vasodilation [62]. Though MR-proANP is not biologically active, it has been demonstrated to be a valuable marker for cardiovascular disease. For example, several studies have demonstrated that it can be used as an early marker of heart failure and hypertrophic cardiomyopathy [63,64,65]. Two studies conducted by Acosta et al. in 2018 [11] and 2021 [34] investigated MR-proANPs association with AAA; however, both studies noted no significant association between the biomarker level and incidence of AAA. 

### 3.2. Hemostasis

A hallmark of abdominal aortic aneurysms, present in almost 80% of patients with AAA, is an intraluminal thrombus (ILT) [66]. These thrombi initiate at the site of the aneurysm due to the vascular endothelial cell damage that activates circulating platelets. The injured vascular wall, as well as activated platelets, further recruit and activate platelets to the local area, creating an early platelet plug [67]. The injured endothelium also exposes subendothelial Tissue Factor (TF), activating the coagulation cascade with the formation of fibrin, which interacts with the platelet plug, forming a stable thrombus. Activated platelets not only contribute to thrombus formation, but also release cytokines, leading to a local inflammatory response, further exacerbating endothelial injury and thrombus formation [68]. Complete aortic occlusion by thrombus formation is not common; however, it can occur, and has a mortality rate of 50% [69]. With hemostatic dysfunction playing an important role in the pathogenesis of AAA, biomarkers related to thrombosis and hemostasis have been investigated as potential predictors of AAA outcomes, as demonstrated by the four markers outlined below. 

#### 3.2.1. Thrombin–Antithrombin (TAT) Complex

Thrombin is an important protein in the coagulation cascade and for platelet activation, allowing for hemostatic plug formation and the cessation of blood loss during injury [70]. After its formation, it is quickly bound to and inhibited by antithrombin, forming the thrombin–antithrombin (TAT) complex. The TAT complex is a stable and relatively less complex protein and is therefore often used as a marker of a hypercoagulable state [71]. A previous study demonstrated a negative relationship between cardiovascular events and TAT complex levels in patients with angina [72]. It is not uncommon to see thrombus formation at the location of aneurysmal disease, and complete occlusions of AAA, though rare, can occur and constitute a medical emergency. Hence, understanding the hypercoagulability of a patient with AAA may be beneficial to preventing thrombus formation and acute occlusions [69,73,74]. One study investigated the relationship between TAT and aneurysmal growth, demonstrating that patients with a greater than 1 µg/mL increase in TAT had a 0.24 mm increase in aortic diameter per year. Patients within the highest quartile of TAT had an odds ratio of 240.02 (21.13, 2725.97) for fast-growing aneurysm. This odds ratio and confidence interval is quite large, however, and must be considered with caution [33]. 

#### 3.2.2. Fibrinogen

During coagulation, activated thrombin binds to and cleaves the soluble protein fibrinogen into its active, non-soluble form, fibrin, which binds to activated platelets to create a platelet plug to prevent blood loss from tissue damage [75]. Fibrinogen is a glycoprotein formed in the liver and released into the plasma, maintaining concentrations around 200–400 mg/dL [76]. Changes in these levels can cause severe disease, with lower levels leading to increased risk of bruising and bleeding, and higher levels associated with hypercoagulable states. These fluctuations can stem from several factors, including congenital disorders, hepatic disease, trauma, and surgery [76]. Fibrinogen levels can influence outcomes post-surgery in patients with cardiac disease; hence, one study investigated perioperative fibrinogen levels on aortic outcomes in patients undergoing EVAR. The authors noted that there was no significant association between perioperative fibrinogen levels and post-operative endoleak, lower limb ischemia, or need for re-intervention. They did note, however, that patients with endoleak had higher median levels of fibrinogen compared to those with no endoleak [9]. 

#### 3.2.3. D-Dimer

D-dimer is a degradation product of fibrin and has been widely used as a marker of the breakdown of hemostatic plugs and thrombi. Specifically, it is used as a diagnostic marker of pulmonary embolism and deep vein thrombosis [77]. In the context of AAA, this may be due to the increased tendency of thrombus formation within aneurysmal sacs. With the fibrinolysis of these thrombi, D-dimer levels would subsequently increase. Several studies have investigated D-dimer as a prognostic marker for AAA. One study demonstrated that a cut-off of >400 ng/mL has an adjusted odds ratio for AAA diagnosis of 12.1 (95% CI 7.1–20.5, *p* < 0.001) [78]. Another study conducted by Sundermann et al. in 2018 demonstrated a similar finding. They noted that patients with >500 ng/mL had 7.19 (95% CI 2.90–17.83) times the odds of having a rapidly expanding aorta (≥10 mm/year) [33]. Moxon et al. wanted to investigated if D-dimer could predict endoleak post-EVAR; however, they noted no significant association [14]. 

#### 3.2.4. Platelet Factor 4 (PF4)

Platelet factor 4 (PF4) is a chemokine released by platelets in response to their activation and inhibits the activity of antithrombin, thereby promoting coagulation [79]. It has also been recently demonstrated that PF4 may play a role in inflammation and immunity by binding to microbial organisms and activating the immune system [80]. Interest in PF4 surged when it was discovered as the antigen targeted in vaccine-induced immune thrombotic thrombocytopenia (VITT) as well as Heparin-Induced Thrombocytopenia (HIT). Heparin binding to PF4 can lead to its immune recognition and degradation [79]. PF4’s role in the progression of AAA is unknown; however, Sundermann et al. in 2018 investigated its association with AAA progression due to its relationship with antithrombin. They noted no significant relationship between AAA growth or any other AAA-related outcome [33]. 

### 3.3. Transport Proteins

The transport of essential nutrients, molecules, and ions is important for almost all physiological processes and is carried out by specific transport proteins. In the vascular system, these proteins are crucial for maintaining homeostasis by transporting essential molecules such as hormones, fatty acids, metals, and proteins throughout the body, including to and from the aortic wall. The dysregulation of some of these transport processes can lead to the accumulation, or lack thereof, of these important molecules. A few proteins related to the transport of ions and other molecules have been implicated in the progression of AAA. 

#### 3.3.1. Ferritin

Ferritin is an important plasma protein responsible for the transport and storage of iron. Ferritin levels are often used as a marker of iron homeostasis, with low levels being associated with hypothyroidism, and iron absorption deficiencies, and high levels with iron hemochromatosis, known as iron overload [81]. Ferritin levels have also been associated with autoimmune conditions including multiple sclerosis, lupus, and rheumatoid arthritis [82]. A study in rodent models has suggested that iron overload may be associated with AAA, and that an iron-restrictive diet was protective against AAA [83]. Moxon et al. [84] investigated this in humans in 2016; however, they noted no significant association between ferritin levels and AAA diagnosis or progression (odds ratio 0.880 (95% CI 0.764–1.015), *p* = 0.078).

#### 3.3.2. Haptoglobin

After the lifespan of erythrocytes, haptoglobin binds to hemoglobin for its recycling to prevent iron depletion and the release of free iron that may cause organ damage [85]. Haptoglobin is also an important acute-phase protein, known to inhibit lymphocyte function and regulate helper T-cells [86]. Haptoglobin has three major phenotypes, Hp 1-1, Hp 2-1, and Hp 2-2, each with their distinct effectiveness in binding hemoglobin and suppressing the immune response [86]. A study in 1990 by Powell et al. [84] demonstrated that Hp 1-1 and Hp 2-1 had a higher activity of elastin hydrolysis compared to Hp 2-2, and patients with Hp 2-2 were higher in age compared to the latter phenotypes, demonstrating slower initiation and progression in the Hp 2-2 AAA phenotype. More recently, Wiernicki et al. investigated if haptoglobin phenotype was associated with AAA expansion. They noted a significantly elevated expansion rate in patients with the H2-1 phenotype of 3.69 ± 2.40 mm/y compared to 1.24 ± 0.79 in the Hp 2-2 group and 1.45 ± 0.68 in the Hp 1-1 group (*p* < 0.0001 for both) [21]. 

#### 3.3.3. Albumin

Albumin is a plasma protein found in the highest abundance in plasma compared to any other protein. Its primary function is to maintain plasma oncotic pressure, to prevent the loss of fluid in the capillaries. It is secreted by the liver and extracted continuously at a rate of 10 gm to 15 gm per day, and is an important marker of liver function [87]. High levels of albumin have previously been attributed to reduced vascular resistance, and hence may play a role in the pathogenesis of AAA [88]. Two recent studies have investigated albumin levels and their prognostic effects on AAA. Interestingly, one study conducted by Wohlauer et al. in 2017 noted that AAA patients with albumin <2.4 g/dL had a significantly elevated 30-day mortality (odds ratio 4.967 (97% CI 1.385–17.814), *p* = 0.025) compared to those with higher albumin [31]. Similarly, Inagaki et al. in the same year noted that those with low albumin (≤3.5 g/dL) had a higher 1-year mortality. They noted that 4.0 g/dL could be used as a cut-off for low albumin, giving an AUC of 0.885, (95% CI 0.821–0.949, *p* < 0.001) and a sensitivity and specificity of 90.0 and 80.3 percent, respectively [89].

### 3.4. Inflammation and Immunity

The formation of AAA is a multifactorial process; however, it has been hypothesized that inflammation plays a central role in the initiation and progression of the disease. It is suggested that AAA may initiate through an initial immune response to an antigen with in the aortic wall, leading to the infiltration of immune cells and the release of cytokines, initiating a cascade of events leading to arctic dilation [90]. Several studies of AAA tissue samples have noted infiltration of inflammatory cells such as macrophages, natural killer cells (NK cells), T and B lymphocytes, mast cells, and neutrophils [91,92]. This occurs due to the release of cytokines and chemokines in response to vascular endothelial damage and platelet aggregation [67,92]. A subtype of AAAs, known as Inflammatory AAA (IAAA), is rare (5–10% of all AAA cases), with a higher mortality, higher likelihood of being symptomatic, and a higher likelihood of surgical complications [93,94,95,96]. Documenting inflammatory markers and determining strong biomarkers of inflammation within the AAA may be beneficial in the early prognostication of the disease.

#### 3.4.1. Elastase

Elastase is a serine protease enzyme which is produced by the acinar cells of the pancreas. Elastase is a digestive enzyme and it is involved in the breakdown of fats, carbohydrates, and proteins to facilitate energy, growth, and cell repair in the body [97]. The aorta is an elastic artery composed of vascular smooth muscle and elastin. One of the known physiological processes related to AAA is the enzyme elastase, which is capable of degrading elastin fibers in the aorta. Destruction of the elastin fibers weakens the structure of the aorta, ultimately leading to aortic dilation [98]. In the study conducted by Wiernicki et al., in 2010, it was demonstrated that there was a positive correlation between AAA expansion and serum elastase activity in the Hp phenotype group (r = −0.29, *p* = 0.0068). The study went on to state that aneurysmal dilatation was linked to significant aortic stiffness or inelasticity with decreased elastin content. The study suggested that elastase is a better and more accurate marker for inflammation and has a stronger association with AAA growth rate [21].

#### 3.4.2. Thioredoxin

Thioredoxin (TRX or TNX) is classified as an oxidoreductase protein which is an enzyme also known as a globular protein that catalyzes an oxidation–reduction (redox) reaction. The small redox protein is found in all organisms and it is essential for life in mammals [99]. In humans, thioredoxins are encoded by two specific genes: TXN and TXN2. A loss-of-function of either of the two genes potentially results in a lethal outcome at the four-cell stage in embryogenesis [100]. Thioredoxin is involved in inflammation, apoptosis, embryogenesis, and cardiovascular disease processes. Increased levels of thioredoxin have been associated with oxidative stress-associated cardiovascular diseases such as myocarditis and AAA and inflammation, which is a major causative factor of cardiovascular diseases. In the study conducted by Martinez-Pinna R. et al., in 2010, the authors localized the release of TRX in the luminal part of the AAA (31 ± 9 ng/mL vs. 9 ± 3 ng/mL, *p* < 0.05), indicating an oxidative stress and/or inflammatory process [101]. They demonstrated that in the training population, the serum TRX levels were increased in AAA patients compared to the healthy control group (50 ± 6 ng/mL vs. 26 ± 3 ng/mL, *p* < 0.05) (2). The authors observed the following: a positive correlation between serum TRX levels with AAA size and expansion (rho = 0.5, *p* < 0.001) and follow-up with AAA samples showed that TRX was positively linked to aneurysmal diameter growth rate (rho = 0.25, *p* = 0.027) [101].

#### 3.4.3. Leukotriene B4 (LTB4)

Leukotriene B4 (LTB4) is the final product of the leukotriene biosynthetic pathway. LTB4 is involved in the recruitment and activation of immune cells such as neutrophils, macrophages, and eosinophils [102]. LTB4 stimulates the production of pro-inflammatory cytokines and functions as a chemokine promoting the migration of macrophages and neutrophils into tissues in response to foreign antigens [103]. The inflammatory process in AAA may be associated with the LTB4 signaling causing the migration of neutrophils and macrophages into the abdominal aortic wall where they release oxidative and proteolytic molecules that facilitate AAA wall destruction, resulting in AAA expansion. In the study completed by Zagrapan B. et al., in 2021, the authors demonstrated neutrophil-derived LTB4 as a significant chemotactic factor released from the AAA site and to date, this novel study is the first to analyze serum LTB4 levels in AAA patients with aneurysmal growth. They authors noted that patients with AAA had higher levels of LTB4 compared to controls; however, there was no association between LTB4 and aortic growth [18].

#### 3.4.4. Complement Factor C3a

C3 is a complement component that is cleaved to C3a and C3b protein fragments catalyzed by C3-convertase [104]. There are three pathways of activating and forming C3a and C3b fragments which are involved in antigen opsonization: the Classical Pathway, the Lectin Pathway, and the Alternative Pathway [105,106]. C3a exhibits the following functions: T-cell activation and survival, angiogenesis stimulation, chemotaxis, mast cell degranulation, and macrophage activation [107]. C3a is unique as it produces both pro-inflammatory and anti-inflammatory responses. C3a induces its innate immune response by binding to the C3a receptor to increase vasodilation by endothelial cell contraction, increasing vascular permeability, mast cell and basophil degranulation of histamine, and degradation of pathogens by neutrophils, macrophages, and eosinophils. C3a also plays a role in chemotaxis for mast cells and eosinophils [107]. In the study conducted by Zagrapan B. et al., in 2021, C3a levels showed a moderate, but statistically non-significant, elevation of median levels among the AAA patient group compared to the healthy control group with the average C3a level at 16.5 μg/mL (IQR = 12.5 μg/mL) vs. 14.3 μg/mL (IQR = 9.1 μg/mL, *p* = 0.156) and LTB4: 30.6 pg/mL (IQR = 33.6 pg/mL) vs. 27.5 pg/mL (IQR = 18.8 pg/mL, *p* = 0.803), respectively [18]. The study contained limited data to support Complement factor C3a’s impact on AAA expansion and its role as a potential biomarker. 

#### 3.4.5. Complement Factor C5a

Complement factor C5 is produced within hepatocytes, and it is also produced by macrophages. C5 is cleaved to C5a and C5b protein fragments by C5-convertase and is essential in innate immunity. C5a is involved as an inflammatory peptide, initiating complement activation, formation of the membrane attack complex (MAC), attraction of innate immune cells, and allergic responses releasing histamine and TNF-a [108]. C5a is a powerful inflammatory mediator and, though not the initiator, high unregulated levels of C5a can be seen in many inflammatory diseases such as sepsis, rheumatoid arthritis, and systemic lupus erythematosus [109]. C5a also functions as an anaphylatoxin by increasing the expression of adhesion molecules on the endothelium, contracting smooth muscles, and increasing vascular permeability [110]. In the study conducted by Zagrapan B. et al., in 2021, the authors stated that elements of the complement system are present in all layers of AAA tissue and they demonstrated the association of the complement system with AAA pathogenesis and neutrophil chemotaxis. The authors found circulating C5a was found to be significantly raised in AAA patients compared to controls (84.5 ng/mL (IQR = 37.5 ng/mL) vs. 67.7 ng/mL (IQR = 26.2 ng/mL, *p* = 0.007, respectively). In addition, the authors also noted serum C5a levels correlated significantly with the increase in AAA maximum diameter over the following 6 months (r = 0.319, *p* = 0.021) [18].

#### 3.4.6. Antiphospholipid Antibodies (aPLs)

Antiphospholipid antibodies (aPLs) are autoantibodies that attack negatively charged or neutral phospholipids or plasma proteins [111]. These antibodies are classified as heterogeneous, and the presence of elevated aPLs is associated with an increased risk for thrombosis and obstetrical complications [112]. They are also know to increase vascular inflammation, leading to the acceleration of the degradation of the extracellular matrix and hence may play a role in the progression of AAA [113]. Recently, a study was conducted to investigate the ability of aPLs to predict the progression of AAA. Although the percentage of aPL levels found in AAA patients was higher than in patients without AAA, the difference was not statistically significant (13.5% versus 6.3%, *p* = 0.189). However, the presence of aPLs at the baseline increased the odds of AAA progression (odds ratio 9.4 (95% CI: 1.0–86.8), *p* = 0.049) [114].

#### 3.4.7. Soluble Urokinase Plasminogen Activator Receptor (suPAR)

Soluble urokinase plasminogen activator receptor (suPAR) is a glycosyl-phosphatidylinositol (GPI)-linked membrane protein consisting of domains presented on many immunologically active cells [115]. Previous studies have shown suPAR to be linked to many inflammatory-related diseases, such as acute liver failure and systemic inflammatory response syndrome. It is also strongly associated with disease severity, readmission, and mortality, establishing suPAR as a prognostic indicator [116]. A recent study investigated the prognostic capability of suPAR in patients with AAA. This study showed no significant difference between AAA patients and patients without AAA (*p* = 0.051). Nevertheless, it was also found that suPAR levels were elevated when tested three years post-operatively in all patients that underwent EVAR (5.5 (95% CI 4.5–6.1) μg/L) in comparison to pre-operative levels [3.3 (95% CI 2.7–3.9) μg/L] (*p* < 0.001) [36]. 

#### 3.4.8. C-Reactive Protein (CRP)

C-reactive protein (CRP) is an acute-phase protein that is evolutionarily conserved and is secreted from the liver in response to a wide variety of cytokines due to trauma, inflammation, and infection. It has a several ligands such as damaged tissues, antigens, and pathogens to mark it for immune destruction [117]. CRP levels are significantly correlated with several cardiovascular diseases and adverse outcomes including coronary artery disease, peripheral arterial disease, stroke, and myocardial infarction [118,119,120]. In this review, a total of eight studies investigated CRP as a prognostic marker for AAA. Three studies demonstrated a correlation between the incidence of AAA and high CRP levels [26,35], with one study noting a hazard ratio of 3.162 (1.690–5.126, *p* = 0.001) [10]. A further three studies noted significant associations between higher CRP levels and aortic growth/expansion rate with odds ratios of 4.3 (95% CI 2.3–7.9) in one study and 6.3 (95% CI 3.1–7.5) in another [13,21,29,37]. One last study noted no significant association between perioperative CRP and risk of endoleak [14]. Though CRP is not quite specific, it could be an important marker to be considered in addition to other markers for prognostication purposes in AAA. 

#### 3.4.9. Citrullinated Histone H3 (CitH3)

Citrullinated histone H3 (CitH3) is a nuclear protein component found in the nucleosome core that organizes the genomic DNA of eukaryotes. Such proteins consist of an unstructured N-terminal tail susceptible to post-translational modifications such as citrullination [121]. This citrullination allows citrullinated histone H3 to be a key player in predicting thromboembolism in cancer patients by detecting neutrophil extracellular trap formation [122]. CitH3 can also induce a robust inflammatory response, potentially promoting the degradation of the extracellular matrix [123]. Recently, a study investigated the presence of histone citrullination as a prognostic factor and inhibition target for AAA. It was found that there was a significant elevation of CitH3 levels in AAA patients in comparison to patients without AAA (362 ng/mL versus 304 ng/mL, *p* = 0.004, respectively). CitH3 was also found to predict AAA growth over six months (*p* = 0.015), with an AUROC equaling 0.707 [23].

#### 3.4.10. Serpin Family A Member 1 (alpha1-AT)

Alpha 1 antitrypsin is a protein encoded by the Serpin Family A member 1 (alpha1-AT) gene. It belongs to the serine protease inhibitor family, also known as serpins. Alpha 1 antitrypsin is produced in the liver and plays a critical role in inhibiting neutrophil elastase, which helps fight infection and prevent lung damage [124]. alpha1-AT’s inhibition of proteases may reduce the breakdown of the extracellular matrix, thereby reducing the rate of AAA growth. A recent study investigated the prognostic effect of Serpin Family A member 1 (alpha1-AT) in AAA. No significant correlation was found between alpha1-AT and AAA wall stress and prospective growth [37]

#### 3.4.11. Tyrosine Kinase Receptor AXL

The tyrosine kinase receptor AXL is a receptor tyrosine kinase that binds to its ligand growth arrest specific 6 (Gas6) and is found in many cells types including monocytes, platelets, endothelial cells, vascular smooth muscle cells, and fibroblasts [125,126,127]. It functions by regulating cell survival, apoptosis, and cell differentiation and platelet aggregation [128,129]. Elevated levels of AXL have been implicated in several cancers due to its oncogenic properties, including hepatocellular carcinoma, renal cancer, and melanoma [130]. Due to it pro-survival influence, and its regulation of endothelial and vascular smooth muscle cells, Ekman et al. [27] investigated serum levels of AXL and if they were correlated with the AAA disease processes. They noted a negative relationship between AAA size and AXL levels, with decreasing levels of AXL being associated with increased AAA diameter with an odds ratio of 0.778 (C0.651–0.933, *p* = 0.0066). 

### 3.5. Kidney Function

Kidney function and failure and AAA have a bidirectional relationship, where renal impairment can contribute to the development of AAA, and conversely, AAA can lead to complications affecting kidney function [131,132]. The prevalence of AAA is 30% higher in those with chronic kidney disease (CKD) [133,134]. Several studies have also demonstrated that renal dysfunction is a major, and not uncommon, risk factor after AAA surgical interventions [135,136,137]. CKD and AAA share several risk factors; however, it is hypothesized that CKD can cause an imbalance in calcium–phosphate metabolism, lead to arterial stiffness, as well as oxidative stress, thereby contributing AAA development [132]. Two studies have investigated renal function markers as prognostic markers in AAA. 

#### 3.5.1. Plasma Cystatin B

Cystatin B belongs to the family-1 of the cysteine protease inhibitor superfamily, and is thought to regulate the activity of cathepsins which are known to cause intracellular protein degradation, antigen processing, and apoptosis regulation. Hence, Cystatin B has been thought to potentially have a regulatory role in AAA [138]. It has also been shown to be upregulated in activated macrophages and translocated to the nucleus to interact with histones and cathepsin L [139]. Mutations in this gene have been implicated in epilepsy [140]. One study investigated plasma cystatin B levels and its association with AAA. The research suggested that patients with elevated levels had a higher likelihood of developing AAA even after controlling for other atherosclerotic vascular diseases, with an odds ratio of 1.526 (95% CI 1.224–1.901, *p* < 0.001). They also noted that each tertile increase in plasma cystatin B constituted a 53.5% increase in the likelihood of needing AAA surgical repair with a hazard ratio of 1.523 (1.251–1.854, *p* < 0.001) [141]. 

#### 3.5.2. Cystatin C 

Cystatin C is an inhibitor of lysosomal and cysteine proteases such as cathepsins and is present in all human bodily fluids, but is found more predominantly in cerebrospinal fluid, semen, and milk [142]. Cystatin C is a well-studied marker of kidney function, as it is freely filtered and reabsorbed by the proximal tubules [143]. Clinical calculators are available to estimate the glomerular filtration rate using Cystatin C levels; however, it is more expensive to test compared to creatinine, and can also be influenced by inflammation, thyroid disorders, and adiposity [144]. Due to its proteolytic activity, AAA has been hypothesized to potentially be associated with AAA. Acosta et al. [34] investigated the association between Cystatin C levels and AAA; however, they noted no significant association between Cystatin C and the incidence of AAA. 

#### 3.5.3. Neutrophil Gelatinase-Associated Lipocalin (NGAL)

Neutrophil gelatinase-associated lipocalin (NGAL) is a protein that is released from both hepatic and immune cells; however, it is most abundantly expressed within the tubular endothelium of the kidney for ion transport [145,146]. In the innate immune system, NGAL functions in iron sequestration to reduce iron availability to pathogens [147]. It is primarily used as a biomarker for kidney damage [148]. Two studies investigated NGAL levels in patients with AAA. Groeneveld et al. noted that though NGAL was higher in patients with ruptured AAAs compared to no rupture, there was no significant association between NGAL and aortic growth [38]. Similarly, a study conducted by Ramos-Mozo et al. noted a significant correlation between NGAL and AAA growth; however, significance was lost after multivariate analysis [149]. 

### 3.6. Cellular Structure

Cellular structure plays an important role in the prognosis of AAA. Changes in cellular structures and their functions can influence the stability of the aortic wall, influence its mechanical properties, leading to the loss of aortic elasticity, and increase the speed of the processes that drive aneurysmal growth [150]. Inflammatory cells, risk factors, and other pathological processes can contribute to the degradation of cellular structural proteins and extracellular matrix components, leading to further aneurysm growth and compromised aortic wall integrity [151]. Understanding these cellular mechanisms in AAA can help identify markers for prognostic purposes, and for the discovery of potential therapeutic targets to prevent aneurysm progression or rupture.

#### 3.6.1. Type IV, Type XVIII Collagen

Collagen is one of the most abundant proteins within mammals and plays a vital structural role in several cells and extracellular matrices in the body, including the vasculature, skin, bones, and tendons [152]. The collagen family is composed of 28 different proteins, all with similar structures, but with distinct features and functions [153]. Collagen type IV is primarily found within the basement membrane of the skin. This collagen has a kinked sheeted structure found within the cutaneous basal lamina, and is more pliable, making it idea for the elasticity of the skin [154]. Type XVIII collagen is a prominent component of the extracellular matrix (ECM) of the liver, but is also found within the ECM of the heart, kidney, and brain [155]. One study investigated circulating levels of type IV and XVIII collagen in patients with AAA, noting that patients with AAA had higher levels of both collagen types compared to controls. When tested by logistic regression, there was no significant association between AAA expansion rate and collagen type IV. There was, however, a significant association between type XVIII and aortic expansion after adjusting for confounding factors [16]. 

#### 3.6.2. Endostatin

Endostatin is the C-terminal fragment of type XVIII collagen, and is produced through the cleavage of this collagen by cathepsin L [156]. Endostatin inhibits endothelial proliferation, and is one of the most potent inhibitors of angiogenesis and tumor growth [157,158]. It has potential implications in AAA due to its ability to inhibit angiogenesis and stabilize the vasculature, as well as its anti-inflammatory effects [159]. One study investigated baseline endostatin levels and aortic expansion rate. The authors noted a significant association between endostatin levels and aortic expansion (*p* = 0.012) [16]. 

#### 3.6.3. Myosin-11

Myosins are known as the prototype of the molecular motor, having the important function of many types of cellular movement and transport. Importantly, myosin’s interaction with actin is vital for muscle contraction [160]. Myosin-11 is a myosin exclusively found within smooth muscle cells, and has been previously noted to be elevated in patients with atherosclerosis is areas of expanding disease due to apoptosis and necrosis of smooth muscle cells [161]. Smooth muscle phenotype changes and apoptosis are hallmarks of AAA, and hence the authors investigated myosin-11 as a marker of AAA. They noted that myosin-11 could be used as a marker of AAA with an AUC of 0.97. It also correlated with changes in AAA diameter (r = 0.513, *p* = 0.021) [162]. 

#### 3.6.4. Microfibrillar-Associated Protein 4 (MFAP4)

Microfibrillar-associated protein 4 (MFAP4) is a protein found within the ECM that is related to the fibrinogen domain family. It is found in elastin-rich areas within several tissues including the heart, arteries, lungs, and skin [163]. In the heart, it is suggested that MFAP4 maintains the aortic and arterial wall integrity by binding collagen and elastin and smooth muscles cells via elastin interactions [164]. As its function is the maintenance of the aortic wall stability, one study investigated the levels of MFAP4 in patients with AAA. The authors noted a significant negative correlation between MFAP4 and aortic growth rate (Spearman’s correlation ρ = −0.14; *p* = 0.0074). The highest tertile of MFAP4 concentrations had a hazard ratio of 0.51 (*p* = 0.001) for the risk of needing surgical repair [22]. 

#### 3.6.5. Matrix Metalloproteinase 9 (MMP9)

Matrix metalloproteinase 9 (MMP9) is a member of matrix metalloproteinase (MMP) family whose primarily role is the breakdown and remodeling of the extracellular matrix in physiological processes such as embryogenesis, angiogenesis, bone remodeling, and wound repair [165]. In the arterial wall, MMPs have been attributed to the breakdown of collagen, elastin, and other structural ECM proteins, and may contribute to aging, atherosclerosis, and hypertension [166]. MMP9 not only degrades ECM proteins; it also triggers inflammatory pathways through the activation of eosinophils and Th2 cells, leading to the release of cytokines and chemokines [167]. MMP9 is a well-known marker of cardiovascular disease, and has been shown to be elevated in atherosclerotic diseases [167]. Three recent studies investigated MM9 as a marker for AAA. In 2010, Speelman et al. demonstrated that there was a significant correlation between AAA growth rate and MMP-9 plasma concentrations (r = 0.32, *p*-value < 0.05) but no significant correlation was found between MMP-9 and AAA wall stress [37]. In 2018, Moxon et al. wanted to determine if MMP could predict endoleak post-EVAR in patients with AAA; however, there was no association between adverse outcomes post-EVAR and perioperative MMP9 [14]. Most recently, however, Georgiadis et al. investigated proMMP levels (pre-activation of the MMP protein). The authors noted that there was an association between perioperative proMMP, noting that patients with endoleak had higher levels of perioperative proMMP-9 compared to those without endoleak (mean difference 8.44 (−19.653 to −1.087), *p* = 0.03). Regression analysis showed proMMP-9 was positively associated with late endoleak (odds ratio 1.055 (1.007–1.106, *p* = 0.025) [19].

#### 3.6.6. TIMP Metallopeptidase Inhibitor 1 (TIMP-1)

TIMP metallopeptidase inhibitor 1 (TIMP-1) is an MMP inhibitor, preventing the degradation of the ECM [168]. A balance between MMP and TIMP activity is necessary to allow for normal physiological activity and preventing pathological conditions [169]. TIMP-1 has been noted to have neuroprotective effects by regulating the blood–brain barrier and reducing glutamate-mediated calcium influx [170]. Two studies have investigated TIMP-1 as a prognostic marker for AAA. The first study conducted in 2010 noted that there was no significant correlation found between TIMP-1 and AAA growth and wall stress [37]. Speelman et al. also investigated TIMP-1 levels in 2010, but noted no significant association between TIMP-1 levels and AAA growth rate and wall stress [37]. More recently, Georgiadis et al. also investigated TIMP-1 levels, and similarly noted that pre-operative TIMP-1 levels could not predict endoleak post-EVAR [19]. 

### 3.7. Hormones and Growth Factors

The abdominal aortic aneurysm disease process facilitates the need for aortic wall remodeling, and often a dysregulation of this process can impair the normal function of the aortic wall. Hormones and growth factors may play a role in the pathogenic involvement of inflammatory cells, diminished proliferation of smooth muscle cells, and the breakdown of cellular structure within the aorta that is a hallmark of AAA [171,172,173]. Six hormones were recently investigated for their association with AAA and adverse aortic events.

#### 3.7.1. Osteoprotegerin (OPG)

As its name suggests, osteoprotegerin (OPG), also known as tumor necrosis factor receptor superfamily member 11B (TNFRSF11B), is a member of the tumor necrosis factor (TNF) receptor superfamily [174]. It has been well characterized for its involvement in bone homeostasis by inhibiting osteoclast formation; however, emerging evidence has demonstrated its functional relevance in many other areas of human physiology and pathology. Critical OPG involvement in vascular biology was first identified in *OPG*^−/−^ mice, which developed arterial and renal calcification [175]. Other functional studies of OPG determined that the absence of OPG leads to extraosseous calcification, and furthermore, vascular dysfunction and atherosclerosis. Serum OPG has been weakly correlated with aneurysm growth rate [176]. One study found that the concentration of OPG in the aortic wall in samples collected from AAA biopsies was correlated with AAA diameter (r = 0.196, *p* < 0.001). The median (interquartile range) OPG concentrations were 16.1 (5.8–28.7), 21.9 (10.2–36.0), and 24.0 (13.5–52.9) ng OPG/mg protein in biopsies removed from patients with <55 mm, 55 to 70 mm, and >70 mm AAAs, respectively (*p* = 0.020) [177]. In this review, however, one study investigated plasma levels of OPG and their ability to predict endoleak post-EVAR, and no significant association was determined between the two [14]. 

#### 3.7.2. Insulin-like Growth Factor I (IGF-I) and Insulin-like Growth Factor II (IGF-II)

Insulin-like growth factors (IGFs) are constitutively expressed in the body and are critical mediators of cellular growth and function. The IGF family consists of insulin, IGF-I, and IGF-II. Structurally, these three peptides share 50% of their amino acid composition [178]. IGF-1 acts in a protective fashion on the vascular endothelium by interacting with high-affinity binding sites, leading to nitric oxide (NO) production, increasing insulin sensitivity, reducing postprandial dyslipidemia, as well as through its other anti-apoptotic and anti-inflammatory pathways [179]. A study published by a group in 2011 found that IGF-1was positively correlated with AAA size (r = 0.23, *p* = 0.016), and AAA expansion rate (r = 0.27, *p* = 0.004). However, ROC analysis was conducted to evaluate the ability of IGF-1 to predict the need for surgery and showed an AUC of 0.63 (0.52–0.73), which demonstrated very moderate predictive capabilities [24]. 

#### 3.7.3. Proneurotensin

Neurotensin is a well-known regulator of satiety, and mitigates fat metabolism and digestion, functioning as a paracrine and endocrine modulator of the cardiovascular system [180]. Proneurotensin/neuromedin is the stable precursor to the tridecapeptide neurotensin and has been proposed as a marker for cardiovascular disease development [26]. An epidemiological study found proneurotensin to be related to cardiovascular disease with a hazard ratio of 1.17 (95% CI, 1.07–1.27, *p* < 0.001) and cardiovascular mortality with a hazard ratio of 1.29 (95% CI, 1.12–1.49, *p* = 0.001), with a significant interaction between proneurotensin and patient sex (*p* < 0.001) on the risk of cardiovascular disease. In women specifically, proneurotensin was related to cardiovascular disease with a hazard ratio of 1.33 (95% CI, 1.17–1.51, *p* < 0.001), total mortality with a hazard ratio of 1.13 (95% CI, 1.01–1.27; *p* = 0.03), and cardiovascular mortality with a hazard ratio of 1.50 (95% CI, 1.20–1.87, *p* < 0.001) [180]. However, a prospective comparison of plasma biomarkers and traditional risk factors found proneurotensin to be associated with incident isolated atherosclerotic disease and not predictive of any adverse AAA events [26]. 

#### 3.7.4. Growth Arrest Specific Gene 6 (Gas6)

Growth arrest-specific gene 6 (Gas6), a member of the plasma vitamin K-dependent proteins, acts as a ligand to the tyrosine kinase receptors Tyro3, Axl, and Mer [27]. Research, primarily in mouse models, suggests the importance of the Gas6/Axl signaling pathway in vascular homeostasis, with notable upregulation at sites of vascular injury. However, these studies using mouse models have led to contradictory results regarding whether Gas6 functions as a pro- or anti-atherogenic agent [181]. Contrary to these results, in vitro studies using human vascular smooth muscle cells has concluded that Gas6 acts in a protective fashion, in part by reducing the pro-inflammatory phenotype expressed by activated VSMCs [182]. Recent studies have highlighted the involvement of the Gas6/Alx system in the pathogenesis of AAA and critical limb ischemia [181]. Another study conducted by Ekman et al. in 2010 noted that plasma Gas6 was significantly associated with AAA size (odds ratio 1.228, 95% CI 1.045–1.442, *p* = 0.0125) [27]. 

#### 3.7.5. Notch1

The Notch signaling pathway is an evolutionarily conserved pathway, responsible for the development and homeostasis of a variety of tissue and organ systems [183]. Mammals have four Notch paralogs, Notch1, Notch2, Notch3, and Notch4. Only Notch1 was found to be investigated for its potential as a biomarker in AAA. Notch1 has long been implicated in the pathology of numerous inflammatory and vascular diseases. In mice, *Notch1*^−/−^ mutants demonstrate defective embryonic arteriovenous differentiation and angiogenic vascular remodeling [183]. Notch1 targeted deletion results in embryonic lethality [184]. In a 2014 study, Wang et al. found that plasma Notch1 and tumor necrosis factor-α-converting enzyme (TACE) were significantly higher in patients with AAA than in controls (Notch: *p* < 0.001; TACE: *p* < 0.0001), citing it as a reliable biomarker for the diagnosis of AAA. Notch1 and TACE levels were not predictive of the aortic diameter in AAA patients. Notch1 was elevated in patients with endoleak post-EVAR compared to those without endoleak (218.6 ± 1.9 pg/mL vs. 195.0 ± 2.1 pg/mL, respectively, *p* < 0.01) and had an AUC of 0.860 (0.791–0.930, *p* < 0.01) [17].

#### 3.7.6. Angiopoietin-2 (Angpt2)

Angiopoeitin-2 (Angpt2) is a key mediator of important vascular processes such as angiogenesis, arteriogenesis, and inflammation [185]. A large-scale proteomic study identified Angpt2 as one of the nine plasma proteins associated with adverse cardiovascular events in patients with stable coronary heart disease. In response to angiogenic factors and inflammatory mediators, endothelial cell Weibel–Palade bodies release Angpt2, acting as a chemoattractant to promote cellular adhesion and the migration of immune cells across the endothelium [186]. In a prospective observational transatlantic study of admitted patients with suspected acute coronary chest pain, Angpt2 was independently associated with all-cause mortality at 24 and 60 month follow-ups in Norwegian and Argentinean populations. Furthermore, in a general-community-based sample in Germany, it was found that high serum Angpt2 levels were predictive of all-cause and cardiovascular mortality [187]. In a study conducted by Golledge el al. in 2013, Angpt2 was significantly higher in patients with AAA compared with controls (3.16 (95% CI 2.51–4.54) ng/mL versus 2.7 (95% CI 2.03–3.72) ng/mL, respectively, *p* < 0.001). Men in the highest quartile of Angpt2 had increased odds of AAA (odds ratio 2.57 (95% CI 1.66–3.97), *p* < 0.001), and increase odds of cardiovascular death (odds ratio 4.12 (95% CI 1.90–8.94), *p* < 0.001). 

#### 3.7.7. Mid-Regional Proadrenomedullin (MR-proADM)

Adrenomedullin (ADM) is a potent vasodilator peptide, responsible for a wide breadth of biological actions in maintaining vascular homeostasis. More specifically, ADM is thought to function in a vasoprotective fashion through mechanisms such as cAMP activation, intracellular Ca^2+^ release, and NO release [188]. ADM is mainly produced by vascular endothelial cells. Due to its short half-life, accurate quantification of ADM is difficult. Instead, mid-regional proADM (MR-proADM), the precursor of ADM, is used as a surrogate biomarker [189]. Finally, MR-proADM has been associated with predicting both carotid artery disease and AAA [34]. 

In a prospective comparison study following 5101 men and women between the ages of 46–73 years of age, MR-proADM was found to be associated with incident isolated atherosclerotic disease (coronary artery disease, atherothrombotic ischemic stroke, carotid artery disease, or peripheral artery disease) (HR 1.17, 95% CI 1.1–1.25) and incident isolated AAA (HR 1.47, 95% CI 1.15–1.88). Elevated MR-proADM’s association with incident isolated clinically significant AAA suggests that its pathogenesis may in part be due to chronic stress on the aortic wall [34]. This study concluded that MR-proADM elevation indicated AAA susceptibility decades before clinical diagnosis [15].

#### 3.7.8. Copeptin

Copeptin is a byproduct of the synthesis of vasopressin, a hormone important in fluid homeostasis, water resorption, blood pressure regulation, and kidney function [190,191]. Copeptin has been demonstrated to be a valuable marker of disease. A recent study demonstrated a high negative predictive value of myocardial infarctions when using copeptin and troponin together [192]. Copeptin has also been used for the diagnosis of diabetes insipidus [193]. One study recently investigated copeptin’s ability to predict incidence of AAA due to its relationship with blood pressure regulation. However, the study demonstrated no association with incidence of AAA (hazard ratio 0.98 (95% CI 0.70–1.39, *p* = 0.092). 

## 4. Discussion

In this comprehensive literature review, 30 articles were demonstrated to investigate prognostic markers of AAA. These 30 articles investigated a total of 45 unique plasma protein biomarkers for the prediction of AAA adverse outcomes including cardiovascular-related outcomes such as stroke, myocardial infarction, and death, as well as aortic-related events such as endoleak, aortic rupture, the need for surgical intervention, and rapid aortic growth. The biomarkers were seen to be involved in several pathways including cardiovascular health, hemostasis, transport proteins, inflammation and immunity, kidney function, cellular structure, and hormones and growth factors. This demonstrates the multi-factorial disease process of AAA, and the importance of investigating multiple pathways for the prognostication of this disease. The inflammatory process of AAA is evident through these results as this pathway has the most prognostic biomarkers. We believe that investigating the inflammatory pathway of this disease further may lead to identifying potential therapeutic targets that could be used to develop more effective treatments. This approach could lead to better management of AAA and possibly slow down or prevent the progression of aneurysmal growth. Additionally, understanding the role of inflammation in AAA could help in early detection and monitoring, ultimately improving patient outcomes.

There has been a recent boom in protein biomarkers as prognostic markers of adverse outcomes that are currently used in a clinical setting for risk stratification and treatment decision purposes. This review demonstrated that biomarkers for AAA could play a similar role in patients’ AAA by assisting in risk stratification. Patients undergoing surgical intervention can be assessed through biomarker analysis, and those at higher risk of adverse events such as stroke, MI, or endoleak could be given the surgical intervention most fitted for that patient. This could allow for more personalized treatment to reduce these complications. Not only could markers be useful for risk stratification, but they could be important for monitoring patients over time, and intervening when the markers reach certain thresholds. 

Machine learning and artificial intelligence have changed the landscape of biomarker usage within the clinical setting due to their ability to determine patterns in data not easily detected with other statistical models. They do, however, require a large amount of data to be more confident in the generalizability of the results. Recent studies have been able to combine multiple plasma markers with clinical characteristics to increase the sensitivity and accuracy of the prediction models. This review has outlined some of the single proteins and their ability to predict outcomes. The key element is the ability to integrate past medical history in combination with these markers to better tailor models to the patient. There are several studies that have used machine learning models to predict adverse outcomes using a combination of imaging, clinical and demographic characteristics, and plasma markers to predict outcomes in patients with AAA [194,195,196,197,198,199,200,201]. For example, a study conducted by Turton et al. used protein levels and clinical characteristics to predict outcomes in patients with AAA. They used hemoglobin, creatinine, amylase, platelet count, and others in combination with other clinical characteristics and demographics to predict mortality post-surgery. They were able to predict postoperative mortality using a neural network with a sensitivity of 86.4% and specificity of 79.3% [195]. In 2016, Monsalve-Torra et al. [197] also investigated the ability of machine learning models to predict hospital mortality using clinical characteristics only. They were able to predict mortality with a sensitivity of 97% and specificity of 95%. These studies provide evidence that machine learning can be used for highly accurate methods to predict outcomes in patients with AAA. There is currently, however, a lack of studies that investigate the ability of biomarkers only to predict outcomes. This may be beneficial for physicians who may not know their patient’s past medical history. Also, no current model has been identified that has demonstrated its robustness thoroughly enough to be accepted for general use in the clinical setting. Further studies using a combination of markers with higher accuracy and clinical features could be conducted to create more robust models to predict a wider variety of adverse events. These models can help physicians make more informed decisions for patients with AAA when deciding between continuing with medical management or pursuing open or endovascular repair options.

There are a few limitations to this study: First, only MEDLINE was used for searching articles. Due to this, some papers may have been excluded due to unavailability in this search engine. To reduce the loss of papers to be included, three individual reviewers individually reviewed each paper to determine its inclusion or exclusion. Secondly, the study was not annotated in PROSPERO. 

## 5. Conclusions

In this comprehensive literature review, the currently available markers for the prognostication of AAA were outlined. Currently, there is no clinically relevant biomarker that can be used for the diagnosis or prognosis of AAA. There is a need for a marker or a panel of markers that can be used to risk stratify patients with AAA to determine those who may progress faster or are at a higher risk of AAA. This review has outlined the currently available data on prognostic markers for AAA. Future studies may investigate new markers or using a combination of these markers to further increase the accuracy of predicting adverse cardiovascular events in this patient population.

## Figures and Tables

**Table 1:** Primary research articles published between January 2010 and August 2023 focusing on prognostic plasma/serum protein biomarkers for abdominal aortic aneurysm.

Title	Authors	Patients and Sample Size	Proteins	Findings
Association Between Perioperative Fibrinogen Levels and the Midterm Outcome in Patients Undergoing Elective Endovascular Repair of Abdominal Aortic Aneurysms.	Kapetanios, D. et al., 2019 [9]	94 patients with AAA	Fibrinogen	There was no association between fibrinogen levels and development of endoleaks, lower limb ischemia, or reinterventions. The median difference between baseline and post-EVAR levels of fibrinogen were higher in patients with endoleak compared to those without endoleak (median −65 mg/dL vs. 15 mg/dL, *p* = 0.04)
Association of C-Reactive Protein-to-Albumin Ratio With the Presence and Progression of Abdominal Aortic Aneurysms.	Cersit, S. et al., 2021 [10]	150 patients with AAA and 100 healthy controls	Serum CRP/albumin ratio	Patients with AAA had higher plasma CRP/albumin compared with controls (*p* < 0.001). Increased CRP/albumin was an independent predictor of AAA with an odds ratio of 3.162 (95% CI: 1.690–5.126, *p* = 0.001)
B-type natriuretic peptide for prediction of incident clinically significant abdominal aortic aneurysm: A population-based prospective study.	Acosta, S. et al., 2018 [11]	5467 patients of which 84 developed AAA	B-type natriuretic peptide (BNP) and mid-regional pro-atrial natriuretic peptide (MR-proANP)	After adjusting for age, sex smoking status, BMI, hypertension, diabetes, and CRP, N-BNP was significantly associated with the risk of AAA with a hazard ratio of 1.29 (95% CI 1.03–1.62). There was no significant association between MR-proANP and AAA
B-type natriuretic peptide predicts postoperative cardiac events and mortality after elective open abdominal aortic aneurysm repair.	Bryce, G. et al., 2013 [12]	106 patients with AAA undergoing open AAA repair	B-type natriuretic peptide (BNP)	BNP levels were higher in patients with post-operative cardiac events (*p* < 0.001), as well as cardiac death (*p* = 0.043). ROC analysis demonstrated a BNP cut-off of 99.5 pg/mL for predicted cardiac events (AUC 0.927) and 448 pg/mL for predicted cardiac death (AUC 0.963)
C-reactive protein predicts aortic aneurysmal disease progression after endovascular repair.	De Haro, J. et al., 2016 [13]	192 patients with AAA undergoing elective EVAR	C-reactive protein (CRP)	Patients within the highest quartile of CRP had the highest rate of aortic expansion, with the rate of expansion increasing with each quartile (−0.5 ± 1%, 3.6 ± 1%, 8 ± 2%, 10 ± 3%; *p* < 0.01). CRP was independently associated with aortic expansion rate (odds ratio 4.3, 95% CI 2.3–7.9)
Circulating biomarkers are not associated with endoleaks after endovascular repair of abdominal aortic aneurysms.	Moxon, J. et al., 2018 [14]	75 patients with AAA undergoing EVAR	Matrix metalloproteinase 9 (MMP9), osteoprotegerin (OPG), D-dimer, homocysteine (HCY), and C-reactive protein (CRP)	Patients with endoleak post-EVAR had elevated levels of HCY compared to those without endoleak (16.0 (11.3–19.2) mol/L versus 12.0 (10.0–14.0) mol/L, *p* = 0.02, respectively). None of the analyzed biomarkers were associated with endoleak after EVAR
Circulating Midregional Proadrenomedullin and Risk of Incident Abdominal Aortic Aneurysm: A Prospective Longitudinal Cohort Study.	Acosta, S. et al., 2018 [15]	5551 patients of which 84 developed AAA	C-reactive protein (CRP) and mid-regional proadrenomedullin (MR-proADM)	After adjusting for age, sex smoking status, BMI, hypertension, diabetes, and CRP, MR-proADM was significantly associated with the risk of AAA with a hazard ratio of 1.28 (95% CI 1.01–1.62)
Circulating Vascular Basement Membrane Fragments are Associated with the Diameter of the Abdominal Aorta and Their Expression Pattern is Altered in AAA Tissue.	Holsti, M. et al., 2018 [16]	615 patients divided into three groups based on aortic diameter: (1) normal aorta ≤ 25 mm, (2) sub-aneurysmal aorta (SAA) 26–29 mm, and (3) AAA ≥ 30 mm. 159 patients followed up	Type IV and XVIII collagen/endostatin	A significant association was found between the baseline levels of type XVIII/endostatin and the aortic expansion rate (*p* = 0.035), but only the initial aortic diameter was significantly associated with expansion of the aorta (*p* = 0.005)
Combining detection of Notch1 and tumor necrosis factor-alpha converting enzyme is a reliable biomarker for the diagnosis of abdominal aortic aneurysms.	Wang, Y. et al., 2015 [17]	110 patients with AAA undergoing EVAR	Tumor necrosis factor-α-converting enzyme (TACE) and Notch1	TACE was elevated in patients post-EVAR with endoleak compared to those without (2376.4 ± 28.1 pg/mL vs. 2094.1 ± 27.3 pg/mL, *p* < 0.01). ROC analysis of the ability of TACE to determine endoleak had an AUC 0.844 (95% CI 0.771–0.918, *p* < 0.01). Notch1 was also elevated in patients with endoleak post-EVAR (218.6 ± 1.9 pg/mL vs. 195.0 ± 2.1 pg/mL, *p* < 0.01) and had an AUC of 0.860 (95% CI 0.791–0.930, *p* < 0.01)
Complement Factor C5a Is Increased in Blood of Patients with Abdominal Aortic Aneurysm and Has Prognostic Potential for Aneurysm Growth.	Zagrapan B. et al., 2021 [18]	40 patients with AAA and 37 controls	Complement factor 3a (C3a), Complement factor 5a (C5a), and Leukotriene B4 (LTB4),	Patients with C3a >101 ng/mL had significantly increased odds of a rapidly expanding AAA (odds ratio 11, 95% CI 1.1–114.1). There was no significant association between Complement factor 5a (C5a) and Leukotriene B4 (LTB4) and AAA expansion
Correlation of Baseline Plasma and Inguinal Connective Tissue Metalloproteinases and Their Inhibitors With Late High-Pressure Endoleak After Endovascular Aneurysm Repair: Long-term Results.	Georgiadis, G. et al., 2019 [19]	72 patients with AAA undergoing EVAR	ProMMP-9, Active MMP-9, and TIMP-1	Patients with endoleak had higher levels of pre-operative proMMP-9 compared to those without endoleak (mean difference 8.44 (95% CI −19.653 to −1.087), *p* = 0.03). Regression analysis showed proMMP-9 was positively associated with late endoleak (odds ratio 1.055 (95% CI 1.007–1.106, *p* = 0.025)
Glycated Hemoglobin Is Associated With the Growth Rate of Abdominal Aortic Aneurysms: A Substudy From the VIVA (Viborg Vascular) Randomized Screening Trial.	Kristensen, K. et al., 2017 [20]	319 patients with AAA of which 61 had diabetes	Glycated hemoglobin (HbA1c)	Patients within the highest tertile of HbA1c had the lowest change in AAA diameter (*p* < 0.001). However, there was no association between AAA diameter and HbA1c (Spearman ρ = −0.09; *p* = 0.13)
Haptoglobin 2-1 phenotype predicts rapid growth of abdominal aortic aneurysms.	Wiernicki, I. et al., 2010 [21]	83 patients with AAA	Haptoglobin phenotypes, elastase serum activity, and CRP serum levels	Hp 2-1 phenotype was an independent predictor of AAA growth (beta coefficient 0.31, *p* = 0.021)
High plasma microfibrillar-associated protein 4 is associated with reduced surgical repair in abdominal aortic aneurysms.	Lindholt, J. et al., 2020 [22]	504 male patients with AAA and 188 controls	Microfibrillar-associated protein 4 (MFAP4)	There was a significant negative correlation between MFAP4 and aortic growth rate (Spearman’s correlation ρ = −0.14; *p* = 0.0074). The highest tertile of MFAP4 concentrations had a hazard ratio of 0.51 (*p* = 0.001) for the risk of needing surgical repair
Histone citrullination as a novel biomarker and target to inhibit progression of abdominal aortic aneurysms.	Eilenberg W. et al., 2021 [23]	63 patients with AAA and 63 matched healthy controls	Citrullinated histone H3 (CitH3)	CitH3 was found to predict AAA growth over six months (*p* = 0.015), with an AUROC equaling 0.707
Insulin-like growth factor I—a novel biomarker of abdominal aortic aneurysms.	Lindholt, J. et al., 2011 [24]	115 patients with AAA	Insulin-like growth factor I (IGF-I) and IGF-II	IGF-I was positively correlated with both AAA size and AAA growth (r = 0.23, *p* = 0.016 and r = 0.27, *p* = 0.004, respectively). ROC analysis to evaluate the ability of IGF-I to predict the need for surgery gave an AUC of 0.63 (95% CI 0.52–0.73)
Lipoprotein(a) and abdominal aortic aneurysm risk: The Atherosclerosis Risk in Communities study.	Kubota, Y. et al., 2018 [25]	13,683 patients, of which 505 developed AAA.	lipoprotein(a) [Lp(a)]	Patients in the highest quintile of plasma Lp(a) had an increased risk of AAA (hazard ratio 1.57 (95% CI 1.19–2.08), *p* = 0.004)
Lp-PLA2 activity and mass for prediction of incident abdominal aortic aneurysms: A prospective longitudinal cohort study.	Acosta, S. et al., 2017 [26]	5551 patients of which 84 patients developed AAA	Lipoprotein-associated phospholipase A2 (Lp-PLA2), proneurotensin, and C-reactive protein	Lp-PLA2 was independently associated with the incidence of AAA (hazard ratio 1.40 (95% CI 1.15–1.72))
Plasma concentrations of growth arrest specific protein 6 and the soluble form of its tyrosine kinase receptor Axl as markers of large abdominal aortic aneurysms.	Ekman, C. et al., 2010 [27]	250 patients with AAA and 141 controls	Growth arrest specific 6 and tyrosine kinase receptor Axl	There was a positive correlation R = 1.228 (95% CI 1.045–1.442, *p* = 0.0125) between a high Gas6/sAx1 ratio and a large size of AAA while a low Gas6/sAx1 ratio did not rule out AAA
Plasma ferritin concentrations are not associated with abdominal aortic aneurysm diagnosis, size or growth.	Moxon, J. et al., 2016 [28]	4024 men of which 293 developed AAA	Ferritin	There was no difference in ferritin levels between patients with AAA and those without AAA (odd ratio 0.880 (95% CI 0.764–1.015), *p* = 0.078)
Prediction of asymptomatic abdominal aortic aneurysm expansion by means of rate of variation in C-reactive protein plasma levels.	De Haro, J. et al., 2012 [29]	435 patients with AAA	C-reactive protein (CRP)	Patients were split into quartiles of CRP, and aortic diameter increased with increasing CRP (35 ± 2, 40 ± 3, 49 ± 4, and 58 ± 5 mm; *p* = 0.01). CRP was independently associated with aortic expansion rate (odds ratio 6.3 (95% CI 3.1–7.5))
Preoperative biomarker evaluation for the prediction of cardiovascular events after major vascular surgery.	Chan, W. et al., 2019 [30]	40 patients with AAA undergoing open AAA repair and 93 control patients without AAA undergoing arterial bypass.	NT-proBNP	Elevated levels of NT-proBNP were an independent predictor of perioperative myocardial infarction with an AUC of 0.81
Preoperative Hypoalbuminemia is a Risk Factor for Early and Late Mortality in Patients Undergoing Endovascular Juxtarenal and Thoracoabdominal Aortic Aneurysm Repair.	Wohlauer, M. et al., 2017 [31]	1089 patients with AAA undergoing fenestrated and branched endovascular aortic repair (FEVAR)	Albumin	Patients with albumin <2.4 g/dL had a higher 30-day mortality (odds ratio 4.967 (95% CI 1.385–17.814), *p* = 0.025)
Preoperative hypoalbuminemia is associated with poor clinical outcomes after open and endovascular abdominal aortic aneurysm repair.	Inagaki, E. et al., 2017 [32]	15,002 patients with AAA undergoing open AAA repair (n = 4956) or EVAR (n = 10,046)	Albumin	Low levels of albumin were associated with increased 30-day mortality and increased length of hospital stay in both patients undergoing open and endovasular repair with AUC of 0.885 (95% CI 0.821–0.949, *p* < 0.001)
Prognostic value of D-dimer and markers of coagulation for stratification of abdominal aortic aneurysm growth.	Sundermann A. et al., 2018 [33]	237 patients with AAA and 115 controls without AAA	Thrombin–antithrombin (TAT), D-dimer, platelet factor 4(PF4)	Patients with D-dimer levels ≥ 500 ng/mL had significantly increased odds of having fast-growing AAA (odds ratio 7.19, 95% CI 2.90–17.83). PF4 concentration was not associated with risk of any outcome. TAT was associated with AAA growth (β = 0.24 mm/y for every 1 μg/mL increase in TAT, 95% CI 0.19–0.29)
Prospective Comparison of Plasma Biomarker and Traditional Risk Factor Profiles for Incident Isolated Atherosclerotic Disease and Incident Isolated Abdominal Aortic Aneurysm.	Acosta, S. et al., 2021 [34]	5381 patients of which 44 developed AAA	Lp-associated phospholipase A2, mid-regional proadrenomedullin, copeptin, mid-regional pro-atrial natriuretic peptide (MR-proANP), N-terminal pro-B-type natriuretic peptide (NT-proBNP), Cystatin C, and proneurotensin	Lp-associated phospholipase A2 activity was significantly associated with incidence of AAA with a hazard ratio of 1.53 (95% CI 1.11–2.11, *p* = 0.009). Mid-regional proadrenomedullin was also significantly associated with incidence of AAA with a hazard ratio of 1.47 (95% CI 1.15–1.88, *p* = 0.002)
Relationship between endothelial dependent vasodilation and size of abdominal aortic aneurysms.	Medina, F. et al., 2010 [35]	30 patients with AAA	C-reactive protein and flow-mediated dilation of the brachial artery (FMDB)	There was a positive correlation between CRP and aortic diameter (R = 0.74 [*p* < 0.001]). Meanwhile, FMDB and aortic diameter had a negative correlation (R = −0.78, *p* < 0.001)
Soluble urokinase plasminogen activator receptor in patients with abdominal aortic aneurysm.	Lingqvist M. et al., 2012 [36]	119 patients with AAA and 36 healthy controls without AAA	Soluble urokinase plasminogen activator receptor (suPAR)	There was no significant association between AAA and suPAR
The influence of wall stress on AAA growth and biomarkers.	Speelman, L. et al., 2010 [37]	18 patients with asymptomatic AAA	MMP-9, TIMP-1, hs-CRP, alpha1-AT	There was a significant correlation between AAA growth rate and MMP-9 plasma concentrations (r = 0.32, *p*-value < 0.05) but no significant correlation was found between MMP-9, TIMP-1, hs-CRP, and alpha1-AT and AAA wall stress
The Potential Role of Neutrophil Gelatinase-Associated Lipocalin in the Development of Abdominal Aortic Aneurysms.	Groeneveld, M. et al., 2019 [38]	39 patients with AAA (13 patients with ruptured AAA and 26 with non-ruptured AAA), and 10 healthy controls	Neutrophil gelatinase-associated lipocalin (NGAL	There was no correlation between NGAL and AAA expansion rate (*p* = 0.34)

Lipoprotein(a) (Lp(a)), homocysteine (HCY), lipoprotein-associated phospholipase A2 (Lp-PLA2), glycated hemoglobin (HbA1c), B-type natriuretic peptide (BNP), thrombin–antithrombin (TAT) complex, platelet factor 4 (PF4), microfibrillar-associated protein 4 (MFAP4), matrix metalloproteinase 9 (MMP9), TIMP metallopeptidase inhibitor 1 (TIMP-1), osteoprotegerin (OPG), insulin-like growth factor I (IGF-I), insulin-like growth factor II (IGF-II), growth arrest specific 6 (Gas6), Angiopoietin-2 (Angpt2), Leukotriene B4 (LTB4), Complement factor 3a (C3a), Complement factor 5a (C5a), antiphospholipid antibodies (aPLs), soluble urokinase plasminogen activator receptor (suPAR), C-reactive protein (CRP), citrullinated histone H3 (CitH3), Serpin Family A member 1 (alpha1-AT), tumor necrosis factor-α-converting enzyme (TACE), and neutrophil gelatinase-associated lipocalin (NGAL).
